# The Minimum Age for School Children

**Published:** 1911-05-13

**Authors:** 


### The Minimum Age for School Children.

The opinions of Miss Cleghorn, President of the National Union of School Teachers, as to the age at which children should enter school, must receive the attention which the status and experience of the lady deserve. Miss Cleghorn agrees that when home conditions are good, it is well that the school age should be postponed, but points out that with many of the children who attend our elementary schools the home conditions are not so good as to make any postponement desirable. Therefore, she argues, children from five years old should be admitted to the infant room. But it must be remembered that where the home conditions are not good for the five-year-old child, they are no better for the child who is still younger. Why, if we are prepared to receive a five year old into school because of the defects of the home, should we not receive a four, three, or two year old, or, for the matter of that, an infant of a year? In short, why should we not make the infant school a crèche—a useful institution but not exactly an educational one? To tell the truth, we think that Miss Cleghorn herself would sympathise with our point of view; for she protests against the entire difference of method which prevails in the infant room from that which obtains in the rest of the school. She realises that to go from a place where lessons are made to take the form of play to another where the discipline is rigid, is hard on the child—and on the teacher also—and on both points many teachers of experience will agree with her. But is the cure not to be found not in any change in the infant room, but in the frank acceptance of the crèche as the daily abiding-place for children under the age of seven whose home conditions are not satisfactory? Is it necessary to provide highly-trained teachers for the play-work of the average child? Is it not rather intelligent nurses that they want? The kindergartner will prepare the child for the teacher, but she may do so more effectively if she does not do so under the same roof—if the child understands that the school is a more severe place than the crèche. The average child will not resent the change. With that bravado which forms a very good working substitute for courage, he will boast to his juniors of what school means, until they, too, will long for the hour when they will experience the strict discipline of the higher institution. Trust the vanity and imagination of youth for that! And for the sorely tried teachers in the junior standards of the elementary school it would be a great help to have pupils who were prepared for, and did not resent, the more severe standards of the schoolroom. Another point, though one that will in all probability not strike a teacher, deserves to be considered. It is usually among the younger pupils in a school that epidemics start—and the effects of epidemics of measles and whooping-cough, not to mention diphtheria, on the physique as well as the education of the nation, is too serious to be ignored. In the crèche, with trained nurses in charge, the first symptoms of these and other ailments would be sooner observed, and perhaps the epidemic might be checked. In the school it is not so—and we have no right to ask that our teachers should be health officials as well. But if the nursery department were definitely separated from the educational department, we might have the full benefit of both. It may be necessary to have a substitute for the home—in many cases it is; but let the substitute be frankly the public nursery; do not let us pretend that it is the school.

